# Anticancer and Antifungal Compounds from *Aspergillus*, *Penicillium* and Other Filamentous Fungi

**DOI:** 10.3390/molecules180911338

**Published:** 2013-09-13

**Authors:** Tanja Thorskov Bladt, Jens Christian Frisvad, Peter Boldsen Knudsen, Thomas Ostenfeld Larsen

**Affiliations:** Department of Systems Biology, Technical University of Denmark, Søltofts Plads, Building 221, DK-2800 Kgs. Lyngby, Denmark; E-Mails: ttb@bio.dtu.dk (T.T.B.); jcf@bio.dtu.dk (J.C.F.); pebok@bio.dtu.dk (P.B.K.)

**Keywords:** anticancer, polyketides, non-ribosomal peptides, terpenoids, fungi, natural products, taxonomy

## Abstract

This review covers important anticancer and antifungal compounds reported from filamentous fungi and in particular from *Aspergillus*, *Penicillium* and *Talaromyces*. The taxonomy of these fungi is not trivial, so a focus of this review has been to report the correct identity of the producing organisms based on substantial previous in-house chemotaxonomic studies.

## 1. Introduction

Filamentous fungi such as *Aspergillus*, *Penicillium* and *Talaromyces* are some of the most incredible chemical factories known today. Accordingly, numerous bioactives such as mycotoxins, antifungal and anticancer agents have been reported in the literature within the last more than 100 years [1]. Despite this many new compounds revealing remarkable new bioactivities are still being discovered, including well-known metabolites such as griseofulvin [2,3,4,5]. This, combined with the fact that large combinatorial libraries have not provided the anticipated number of new chemical entities explains why the field of natural products is currently assuming new prominence. Thus, natural products are still used as scaffolds for synthetic organic chemistry, although nowadays primarily for designing smaller and more focused diversity oriented synthesis-derived libraries [6].

It has been estimated that approximately 1.5 million or likely as many as 3 million fungal species exist on Earth, of which only around 100,000 species have been described so far [7]. A multitude of new species are likely to be discovered from diverse habitats, such as tropical forest plants and soils, associated to insects and in the marine environment. In addition to untapped biodiversity recent sequencing of complete fungal genomes has revealed that many gene clusters are silent, suggesting the possibility for many more compounds [8]. Despite several efforts to stimulate such pathways using epigenetic modifiers [9,10], it is evident that we still do not know the full biosynthetic potential even of well studied model organisms such as *Aspergillus nidulans*, altogether strongly indicating Nature’s potential as source of new promising bioactive small molecule scaffolds.

The renewed interest in natural product discovery is further enhanced with the new strategies and methodologies for fast dereplication that have been developed within the last decade. Thus chromatographic and spectroscopic methods are combined with database searching in for example Antibase, which is a comprehensive database for natural products from microorganisms [11]. The performance of mass spectrometers is continuously improving, including easy access to both positive and negative ionization spectra even during fast ultra-high-performance liquid chromatography (UHPLC). Altogether the use of accurate mass measurements for dereplication of unknown compounds reduces the number of predicted elemental compositions, ensuring that database searches are conducted with the fewest possible candidates [12]. The use of UV spectral information is often important in the dereplication process for prioritizing between the MS-generated candidates, as well as for UV-guided discovery of novel compounds [13,14].

This article reviews anticancer and often also antifungal natural products primarily produced by *Aspergillus*, *Penicillium* and *Talaromyces*. For practical reasons the compounds have been grouped into major biosynthetic classes, according to the biosynthetic origin of the core part of the structures, despite that many compounds are actually of mixed biosynthetic origin (e.g., prenylated). This review is broader in scope than other recent reviews only focusing on endophytes [15,16], and a strong focus has been on giving the correct identity of the species reported in the literature, where many may previously have been misidentified or names have been changed according to the 2011 Amsterdam Declaration on Fungal Nomenclature [17].

## 2. Polyketide-Derived Anticancer Compounds

Polyketides represents one of the major classes of natural products of which many are biological active [1]. Today much is known about the enzymes involved in the biosynthesis of a huge diversity of both non-reduced (aromatic), partly reduced and highly reduced polyketides [8].

**Figure 1:**
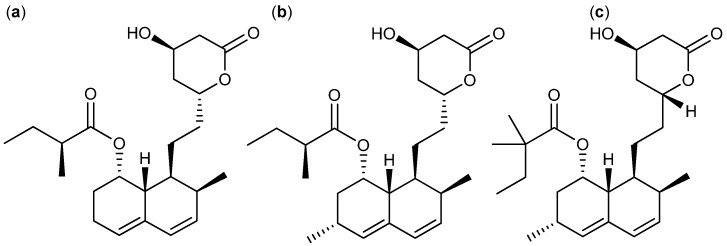
Statins: (**a**) Compactin, (**b**) Lovastatin and (**c**) Simvastatin.

Some famous fungal polyketides with anticancer activity belong to the statin family. The statins are well known cholesterol synthesis inhibitors that are used in clinical treatment of hypercholesterolemia and cardiovascular diseases [18,19,20,21]. Moreover, members the statin family are known to have antifungal properties against *Aspergillus* spp. and *Candida* spp. [18,22,23,24]. The statin family includes a long list of both natural and synthetic compounds, for example the naturally derived compactin, lovastatin and pravastatin. The statin structure is based on a dicyclohexene ring system connected to a dicyclohexene ring system connected to a side chain with a closed lactone ring or an open acid form [21]. The compactins are primarily produced by *P. solitum* and *P. hirsutum* [20,25] (first misidentified as *P*. *brevicompactum* [18] and a fungus identified as *P*. *citrinum* [19]). Another group of statins that has an extra methyl group attached on the dicyclohexene ring system are produced by *A. terreus* [26] and *Monascus* spp. [27]. Several *in vitro* activities of the statins have been published throughout the years. Reports showed that compactin (Figure 1a) inhibited acute myeloid leukemia (AML) cells with a full inhibitory concentration (IC_100_) of 2.6 µM [28]. The analogs lovastatin (Figure 1b) and simvastatin (Figure 1c) have been shown to be even more potent.

Lovastatin and the synthetic simvastatin selectively inhibited colony growth of primary AML cells with 75%–95% effectiveness. No effect was seen on normal bone marrow [29]. The more recent reported activities includes reduction of proliferation by lovastatin in four lung cancer cell lines with median inhibitory concentration (IC_50_) values between 1.5 and 30 µM [30]. In 2010 it was shown that lovastatin induced apoptosis in ten ovarian cancer cell lines tested, with IC_50_ values between 2 and 39 µM [31], and recently lovastatin was found to inhibit breast cancer MCF-7, liver cancer HepG2, and cervical cancer HeLa cell lines with IC_50_ values of 0.7, 1.1 and 0.6 µg/mL, respectively [32]. Simvastatin inhibited two lung cancer, three melanoma, and four breast cancer cell lines with IC_50_ values between 0.8 and 5.4 µM and induced apoptosis with reduced tumor growth in hepatic cancer cells [33,34]. Encouraged by these results simvastatin has entered clinical trials as an anticancer drug [35].

**Figure 2:**
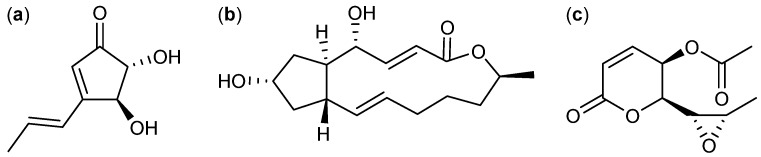
Small polyketides: (**a**) Terrein, (**b**) Brefeldin A, and (**c**) Asperlin.

The three small polyketides terrein, brefeldin A, and asperlin are examples of well-known metabolites that a couple of decades after they were discovered were shown to exhibit novel anticancer activities. The small antifungal [36] polyketide terrein (Figure 2a) produced by *A. terreus* has been known since 1935 [37]. Almost 80 years later it was found that terrein inhibits breast cancer by induction of apoptosis with an IC_50_ value of 1.1 nM in MCF-7 cell line. That makes terrein 100-fold more potent than taxol against this cell line. Additionally terrein was found active against pancreatic and liver cancer cell lines PANC-1 (IC_50_ 9.8 µM) and HepG2 (IC_50_ 66.8 µM) [38].

Brefeldin A (Figure 2b), another small antifungal [39,40] polyketide isolated in 1958 from *P. brefeldianum* [41] was identified almost 40 years later as inducer of apoptosis in leukemia (HL-60 and K-582), colon (HT-29), prostate (DU-145), cervical (KB and HeLa), breast (MCF-7 and BC-1), and lung (SPC-A-1 and NCI-H187) cancer cell lines [42,43,44,45]. The inhibiting effect of brefeldin A was demonstrated with IC_50_ values 35.7 nM (HL-60), 32 nM (KB), 6.4 nM (HeLa), 7.1 nM (MCF-7), 40 nM (BC-1), 6.3 nM (SPC-A-1), and 110 nM (NCI-H187) [44,45].

Asperlin (Figure 2c) was isolated from *A. nidulans* in 1966 [46]. In 2011 it was discovered that asperlin reduces cell proliferation and induce G_2_/M cell cycle arrest in the human cervical carcinoma HeLa cell line [47].

**Figure 3:**
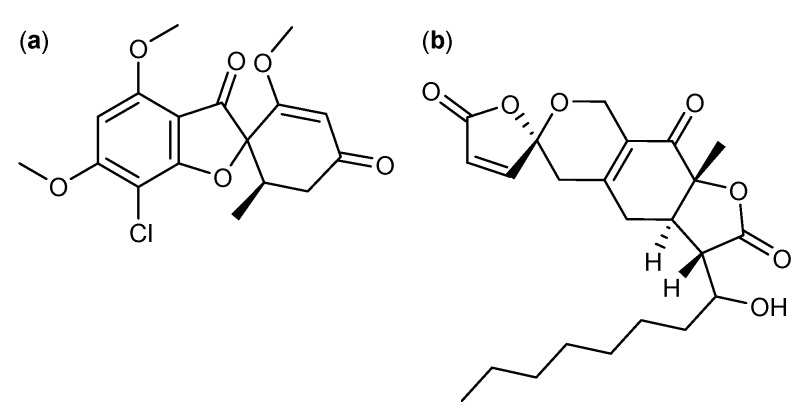
Spiro compounds: (**a**) Griseofulvin, and (**b**) Sequoiamonascin A.

Filamentous fungi are known to produce anticancer polyketides with different spiro ring structures. One of the more well-known is the antifungal [48,49] compound griseofulvin (Figure 3a) from *P. griseofulvum* [50]. Griseofulvin was introduced commercially in 1965 and first considered for cancer treatment in 1973 [49,51]. Later it was shown to induce cell proliferation and mitosis in the human cervical cancer cell line HeLa with an IC_50_ value of 20 µM, as well as it inhibits centrosomal clustering in human squamous cancer SCC-114 cell line with an IC_50_ value of 35 µM [2,3]. The synthetic analog GF-15 increased the inhibitory effect of centrosomal clustering in SCC-114 cells 25-fold with an IC_50_ value of 0.9 µM [5]. It was further shown that combined treatment of griseofulvin and the cancer chemotherapeutic agent nocodazole *in vivo* improved the effect of nocodazole and arrested tumor growth in mice infected with COLO 205 tumors [4].

Other examples of fungal anticancer polyketides with spiro ring structures are sequoiamonascin A (Figure 3b) and B from *A. parasiticus* [52]. Sequoiamonascin A demonstrated selective cytotoxicity against six leukemia cell lines and two melanoma cell lines with a median growth inhibitory (GI_50_) log_10_ value of -6.00 [52]. Furthermore, sequoiamonascin A shows cytotoxic activity against breast cancer MCF-7, lung cancer NCI-H460 and central nervous system (CNS) cancer SF-268 cell lines where cell growth can be reduced to 1%–2% when treated with 10 µM sequoiamonascin A [52].

**Figure 4:**
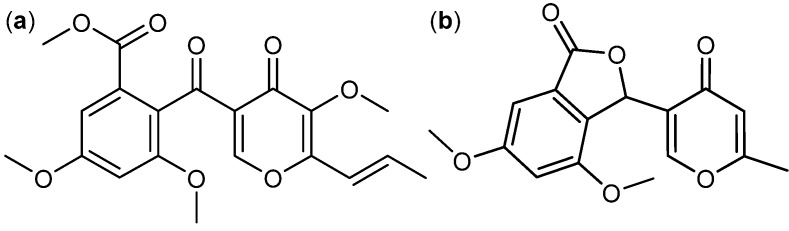
γ-Pyrones: (**a**) 3-*O*-Methylfunicone and (**b**) Penisimplicissin.

A group of fungal γ-pyrones were found to have activity against cancer. An example of these is the antifungal [48,49] 3-*O*-methylfunicone (Figure 4a) produced by *T. pinophilus* (originally published as *P. pinophlium*) [1,48]. It induced apoptosis and affects cell proliferation in various cancer cell lines including HeLa, MCF-7, A-375P and A-375M [50,51,52,53]. Another structurally related compound is penisimplicissin (Figure 4b) isolated from *T. pinophilus* (originally published as *P. simplicissimum*) [54]. It displayed selective cytotoxicity towards leukemia HL-60 and CCRF-CEM cell lines with log_10_ GI_50_ −6.7 and −5.8, respectively [55].

**Figure 5:**
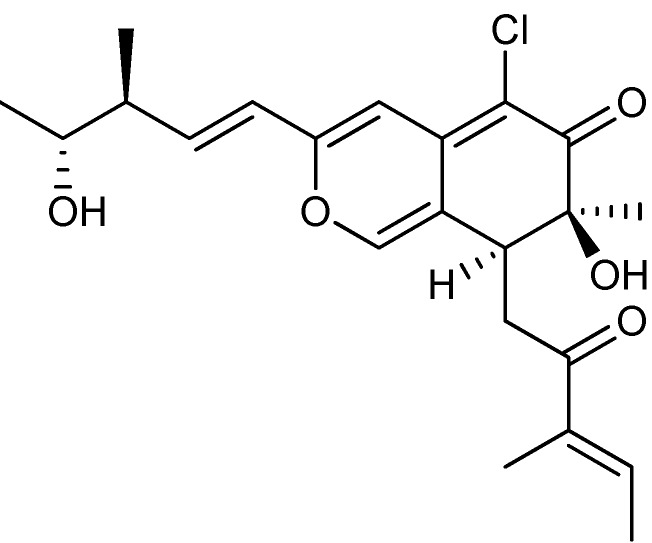
11-Epichaetomugilin I.

A group of more than 20 closely related azaphilones, namely the antifungal [56] chaetomugilin family, has been isolated within the last five years from marine fish-derived *Chaetomium globosum* [57,58,59,60,61,62,63,64]. The chaetomugilins C, I, P and 11-epichaetomugilin I (Figure 5) showed significant inhibition of two human leukemia P-388, HL-60 and one murine leukemia L-1210, as well as human cervical cancer cell line, KB with 11-epichaetomugilin I as the more potent with IC_50_ values of 0.7 µM (P-388), 1.0 µM (HL-60), 1.6 µM (L-1210), and 1.2 µM (KB) [62].

**Figure 6:**
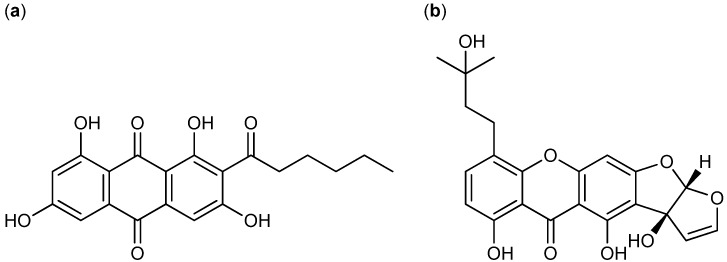
(**a**) Norsolorinic acid and (**b**) Austocystin D.

The fungal metabolite norsolorinic acid (Figure 6a) with a tricyclic structure is produced by *A. parasiticus* and *A. nidulans* [65,66,67,68]. Norsolorinic acid selectively induced cell cycle arrest in G_0_/G_1_ phase of the cell cycle and consequently induced apoptosis in human bladder cancer T-24 and human breast cancer MCF-7 with IC_50_ values of 10.5 and 12.7 µM, respectively [67,68].

Austocystin D (Figure 6b) was isolated from *A. pseudoustus* [69] in 1974 (originally misidentified as *A. ustus* [70]). Austocystin D was shown to selectively inhibit growth of human colon carcinoma LS174T cells in mice and tumor cell lines that overexpress the multidrug resistance-associated protein [71]. Additionally, austocystin D inhibited a number of cancer cell lines: SR (leukemia, GI_50_ 16 nM), U-87 (brain, GI_50_ 4946 nM), MCF-7 (breast, GI_50_ < 1 nM), MDA-MB-231 (breast, GI_50_ 549 nM), PC-3 (prostate, GI_50_ 3 nM), SW-620 (colon, GI_50_ 27 nM), HCT-15 (colon, GI_50_ 42 nM), and MX-2 (uterine, GI_50_ 3358 nM) [72]. Although, displaying diverse activities towards many types of cancer lines, austocystin D never entered clinical trials due to a low safety window [71].

**Figure 7:**
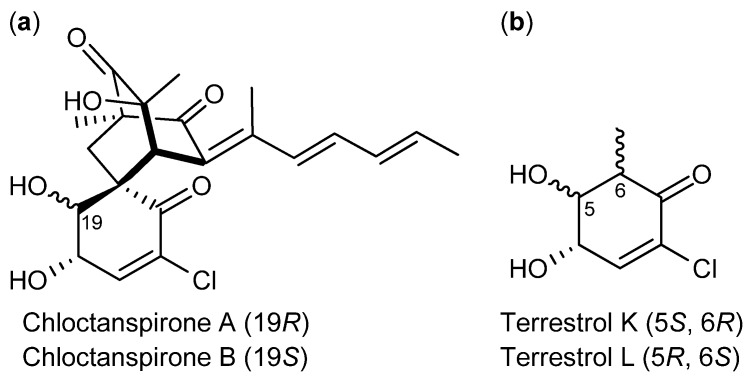
(**a**) Chloctanspirone A, B and (**b**) Precursors terrestrols K, L.

The two epimers chloctanspirone A and B (Figure 7a) are probably produced by *P. chrysogenum* or *P. rubens* [73,74] (originally published incorrectly as *P. terrestre* [75]). Chloctanspirone A and B were the first chlorinated sorbicillinoids isolated from a natural source and the first to be identified with their very unique ring structure. Chloctanspirone A was the more active analog and inhibited human leukemia HL-60 and lung cancer cell line A-549 cell lines with IC_50_ values of 9.2 and 39.7 µM, respectively [76]. Chloctanspirone B however, only showed moderately or no activity against the same cell lines. Interestingly, the two precursors terrestrols K and L (Figure 7b) that contain the epicenter, which dissociates chloctanspirone A from B were inactive. This points to the conclusion that the cyclohexenone moiety has an impact on the activity though it is not the pharmacophore [76].

**Figure 8:**
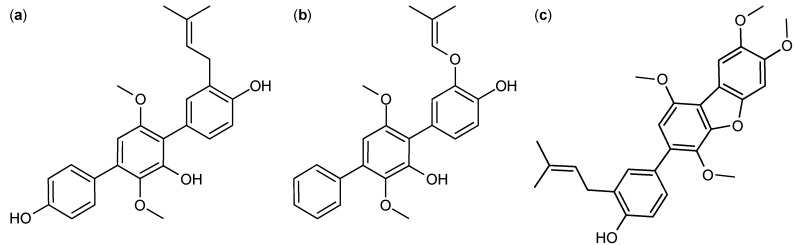
Prenylterphenyllins: (**a**) Prenylterphenyllin A, (**b**) 4′′-Deoxyisoterprenin, and (**c**) Prenylcandidusin B.

A group of around 20 *p*-terphenyls were isolated from *A. candidus* and found to be cytotoxic against the human cervical cancer cell line HeLa [77]. Thirty years later a new group of prenylated-*p*-terphenyls, called prenylterphenyllins (Figure 8a), terprenins (Figure 8b), and prenylcandidusins (Figure 8c) were isolated from *A. candidus* and *A. taichungensis* [78,79]. The activity of these compounds were studied against lung cancer cell line A-549, human leukemia cell lines HL-60 and P-388, and human epidermoid cancer cell lines KB3-1 [78,79]. Prenylterphenyllin A was found to be the more active against A-549 and HL-60 with IC_50_ values of 8.3 and 1.5 µM, respectively [79], whereas 4′′-deoxyisoterprenin displayed higher activity towards KB3-1 with an IC_50_ value of 6.2 µM [78], and finally prenylcandidusin B showed higher activity against P-388 with an IC_50_ value of 1.6 µM [79].

**Figure 9:**
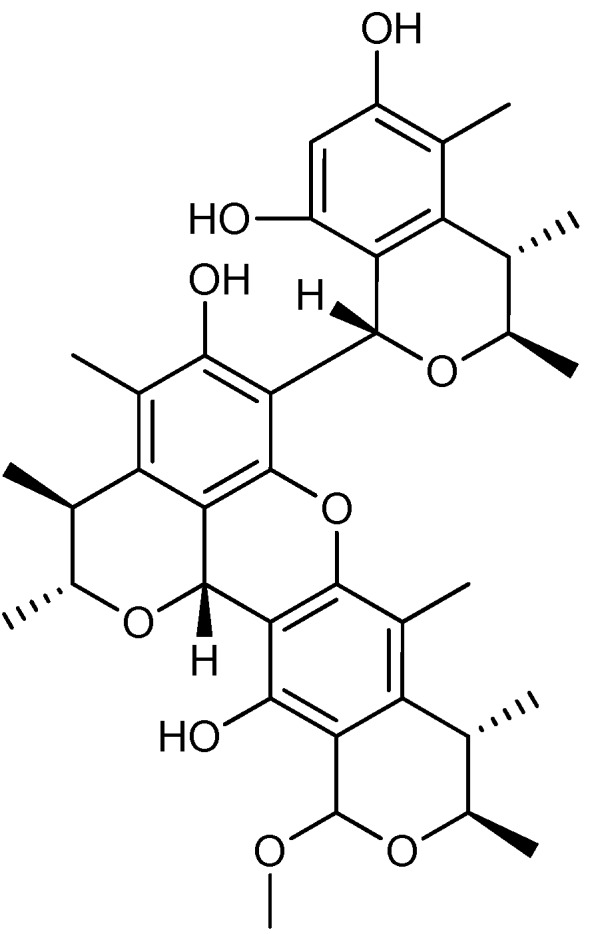
Tricitrinol B.

In 2011 five new di- and tricitrinols were added to the known citrinin family from *P. citrinum* [80]. All five of them showed cytotoxic activity against human leukemia HL-60, colon cancer HCT-116, and cervical cancer KB cell lines. The more potent was tricitrinol B (Figure 9), with IC_50_ values of 3.2, 4.8 and 3.9 µM, respectively [80].

**Figure 10:**
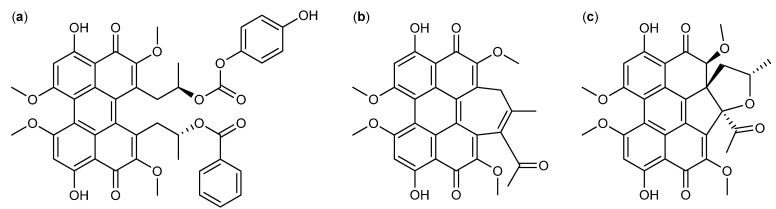
Perylenequinones: (**a**) Calphostin C, (**b**) Hypocrellin B and (**c**) Phaeosphaerin B.

The class of perylenequinones has been used for centuries in Chinese herbal medicine, but fungal production of several perylenequinones has likewise been reported [81]. The calphostins were isolated from *Cladosporium cladosporioides* and shown to inhibit cervical cancer HeLa-S_3_ and breast cancer MCF-7 cell lines. Most potent was calphostin C (Figure 10a) with IC_50_ values of 0.23 and 0.18 µM, respectively [82]. Furthermore, calphostin C induced apoptosis in acute lymphoblastic leukemia [83]. The hypocrellins, another branch, of the perylenequinone family was isolated from *Hypocrella bambusae*, originally published incorrectly as *Shira bambusicola* [84,85]. Hypocrellin D was one of the more potent analogs and inhibited liver (Bel-7721) and lung (A-549 and Anip-973) cancer cell lines, with IC_50_ values, 1.8, 8.8, 38.4 µg/ml, respectively [85]. Additionally, the hypocrellins had a photodynamic effect on a wide range of tumor cell lines. Due to their insolubility in water several analogs have been synthesized to improve the pharmacokinetics and drug delivery. Two of the more successful analogs were carboxylate salt derivatives of hypocrellin B (Figure 10b) with increased water solubility and activity against human breast cancer MCF-7 when photodynamic therapy was used [81,86]. In 2012 a new group of perylenequinones called the phaeosphaerins were isolated from *Phaeosphaeria* sp. with phaeosphaerin B (Figure 10c) as the more potent against prostate PC-3, DU-145, LNCaP cancer cell lines with IC_50_ values of 2.4 µM, 9.5 µM and 2.7 µM, respectively [87].

## 3. Nitrogen-Containing Anticancer Compounds

Fungal nitrogen containing compounds represent another large group of natural products, of which many have famous bioactivities. In general these types of compounds incorporate amino acid building blocks into often complex heteroaromatic compounds such as diketopiperazines, quinazolines and benzodiazepines [1]. Often these compounds are referred to as alkaloids, due to their basic nature, when containing primary, secondary or tertiary amine functionalities. However, compounds only containing amide bonds, that are essentially neutral, are often also referred to as alkaloids. In recent years it has become clear that many nitrogen containing compounds are biosynthesized by multifunctional enzymes (non-ribosomal peptide synthases, NRPS) with modular arrangements comparable to that seen for some polyketide synthases. In such contexts these compounds are usually referred to as non-ribosomal peptides (NRPs) even though they are also called alkaloids [88]. In the following section no attempt has been made to differentiate between the different naming of the numerous amino acid-derived metabolites.

**Figure 11:**
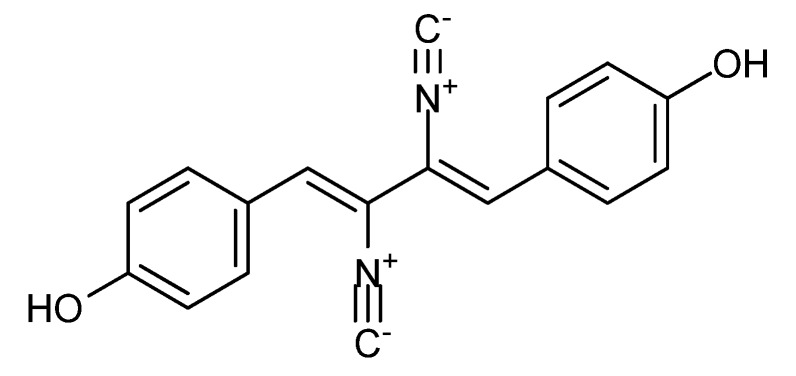
Xanthocillin X.

The antifungal [89] xanthocillin X (Figure 11) was first isolated from *Dichotomomyces albus* ascribed to *D. cejpii* [90], but is produced by *P. chrysogenum* as well [1]. In 1968 xanthocillin X was found active against a Ehrlich ascites carcinoma-mouse strain with median lethal dose (LD_50_) of 40 mg/kg [91]. Approximately 40 years later it was shown that xanthocillin X inhibited leukemia K-562, human cervical cancer HeLa, breast cancer MCF-7 and MDA-MB-231, liver cancer HepG2, lung cancer NCI-H460, and prostate cancer DU-145 cell lines with IC_50_ values between 0.4 and 12 µg/ml [89,92]. Two structural analogs named BU-4704 and xanthocillin X di-methoxy were found more potent than xanthocillin X. Instead of the two hydroxy groups BU-4704 contains a methoxy and a sulfonic acid group and xanthocillin X di-methoxy contains two methoxy groups. BU-4704 inhibited human colon HCT-116 and murine melanoma B16-F10 with IC_50_ values of 0.6 and 4.3 µg/mL, respectively [93]. Xanthocillin X di-methoxy inhibited HepG2, MCF-7 and KB cancer cell lines with IC_50_ values of 0.18, 0.38 and 0.44 µg/mL, respectively [94].

**Figure 12:**
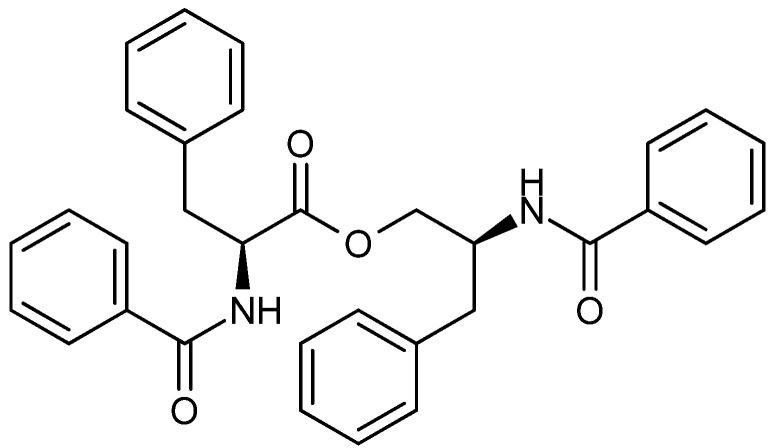
Asperphenamate.

Asperphenamate (Figure 12) was first isolated from *A. flavipes* [95] and later found to be produced by several of *Penicillium* spp. as well [96]. Asperphenamate displayed moderate cytotoxic activity against several cancer cell lines [97]. Due to the low activity and a low water solubility asperphenamate was used as a lead for synthetic structurally isomers. It was found that the (*R,S*) stereoisomers were more potent than the (*S,R*) stereoisomers [98]. The more active asperphenamate derivate was N-benzoyl-O-(N′-(1-benzyloxycarbonyl-4-piperidiylcarbonyl)-D-phenylalanyl)-D-phenyl- alaninol (BBP). BBP was approx. 20-fold more active than asperphenamate against breast cancer (MCF-7, T47D and MDA-MB231), hepatic (BEL-4702), lung (A-549), and cervical (HeLa) cancer as well as leukemia (HL-60) cell lines, with IC_50_ values between 3.0 and 18.3 µM [99].

**Figure 13:**
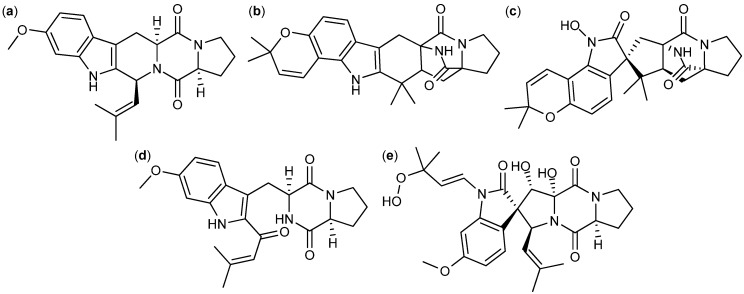
Tryptophan/proline diketopiperazines: (**a**) Fumitremorgin C, (**b**) Stephacidin A, (**c**) Notoamide A, (**d**) 18-Oxotryprostatin A and (**e**) Spirotryprostatin E.

Cyclic di-peptides called diketopiperazines are in general known as cell cycle inhibitors of the G2/M phase [100]. Tryptophan/proline diketopiperazines covers the compound groups fumitremorgins, stephacidins, notoamides, tryprostatins, *etc*. This group of compounds is produced by a long range of *Aspergilli* [101,102,103,104,105,106,107,108,109,110]. The fumitremorgins are produced by *A. fumigatus* and *A. fischeri* [101,102,103,111]. Fumitremorgin C (Figure 13a) was found active against human leukemia P-388 with an ED_50_ value of 3.9 µg/mL and the analog 12,13-dihydroxyfumitremorgin C has antiproliferative effects on human leukemia U-937 and human prostate cancer PC-3, with IC_50_ values of 1.8 and 6.6 µM, respectively [111,112]. Furthermore fumitremorgin C was specifically and potently cytotoxic against multi-drug resistant breast- and colon cancer [113,114].

Two other tryptophan/proline diketopiperazines are stephacidin A (Figure 13b) and B produced by *A. ochraceus* and *A. westerdijkiae* [106,107]*.* Stephacidin B is the dimeric form of stephacidin A and was approximate 10-fold more potent. Stephacidin B exhibited inhibitory effect on prostate, ovarian, colon, breast and lung cancer cell lines with IC_50_ values between 0.06 and 0.4 µM [106].

The notoamides (Figure 13c) produced by *A. amoenus* are close analogs to the stephacidins, but with a spiro ring structure [115]. Contrary to the stephacidins the notoamides only showed low to moderate inhibition of two leukemic cell lines with IC_50_ values 22–52 µg/mL [116].

Finally, the tryprostatins (Figure 13d) and spirotryprostatins (Figure 13e) isolated from *A. fumigatus* where found active against lung (NCI-H-522 and A-549), breast (MCF-7) and prostate (PC-3) cancer as well as leukemia (HL-60 and MOLT-4). The more potent analogs were Ds1-tryprostatin B with GI_50_ values of 15.8 µM (NCI-H-522), 15.9 µM (MCF-7), and 11.9 µM (PC-3) and 18-oxotryprostatin A with an IC_50_ value of 1.3 µM (A-549) [108,110]. Spirotryprostatin E was the more potent of the spirotryprostatins, with IC_50_ values of 3.1 µM (MOLT-4), 2.3 µM (HL-60) and 3.1 µM (A-549) [109].

**Figure 14:**
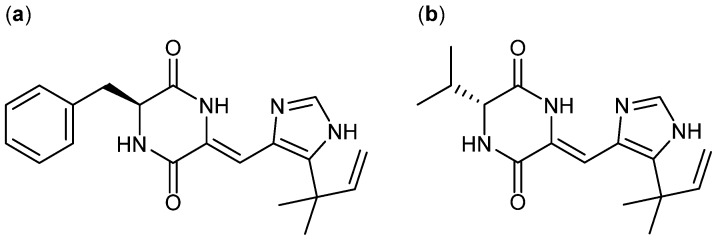
(**a**) Phenylahistin and (**b**) Aurantiamine.

Other diketopiperazines originating from histidine are phenylahistin (Figure 14a) and aurantiamine (Figure 14b). Phenylahistin was first isolated from *A. ustus* in 1997 as a mixture of enantiomers [117]. (−)-Phenylahistin was more active than (+)-phenylahistin against dermal (A-431), lung (A-549), ovary (HeLa), leukemia (K-562 and P-388), breast (MCF-7), CNS (TE-671) and colon (WiDr) cancer cell lines with IC_50_ between 0.18 and 3.7 µM [118]. A synthetic analog plinabulin (NPI-2358) displays very potent activity against human prostate carcinoma cell line DU-145 and has now entered phase II clinical trials [119]. Recently, more than 60 synthetic analogs of plinabulin have been designed and synthesized. The more active analog with a benzoyl group coupled on the phenylalanine unit was 10-fold more active than plinabulin with an IC_50_ value of 1.4 nM [119]. The activity of phenylahistin was also compared to the diketopiperazine aurantiamine and other synthetic analogues [120], originally isolated by Larsen *et al*. [121].

**Figure 15:**
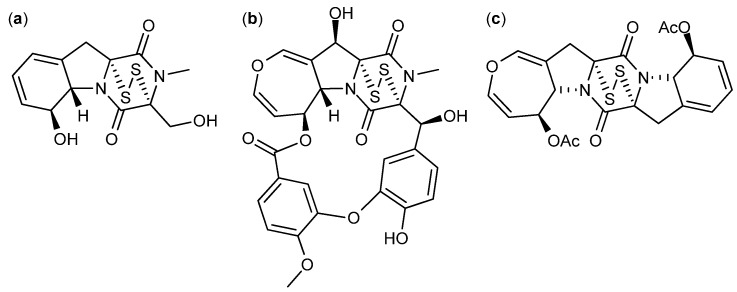
Diketopiperazines with a di-sulfide bridge: (**a**) Gliotoxin, (**b**) Emestrin A and (**c**) Acetylapoaranotin.

Another group of diketopiperazines with anticancer activity contain a di-sulfide bridge in the diketopiperazine ring. One of them the antifungal, immunosuppressive and antimicrobial compound gliotoxin (Figure 15a) that was isolated from *A. fumigatus*, and *D. cejpii* [122,123,124]. Already in 1947 the anticancer activity of gliotoxin was suggested and in 2004 it was found that gliotoxin was a very potent inhibitor of six breast cancer cell lines with IC_50_ values between 38 and 985 nM [125,126]. In 2012, gliotoxin was found active against human leukemia U-937 and human prostate cancer PC-3 cell lines with IC_50_ values of 0.2 and 0.4 µM, respectively [112].

Another diketopiperazine with a di-sulfide bridge is the antifungal [127] compound emestrin A (Figure 15b) that was isolated from *Emericella striata*, now called *A. striatus* [128]. Emestrin A inhibits human leukemia (HL-60) with an IC_50_ value of 83.5 nM [129]. Eight structural analogs of emestrin A were isolated from *Cladorrhinum* sp. and found to have strong antiproliferative effects on the human prostate DU-145 cancer cell line with an IC_50_ value of 9.3 nM for the more potent emestrin C. Furthermore, it was proven that the activity decreased when the macro cyclic ring was opened and the polysulfide bridge in the diketopiperazine was absent [130].

Recently, a new diketopiperazine with a di-sulfide bridge named acetylapoaranotin (Figure 15c) produced by a marine *Aspergillus* sp. was discovered and found active against colon (HCT-116), gastric (AGS), lung (A-549), and breast (MCF-7) cancer cell lines with IC_50_ values of 13.8, 12, 2 and 10 µM, respectively [131].

**Figure 16:**
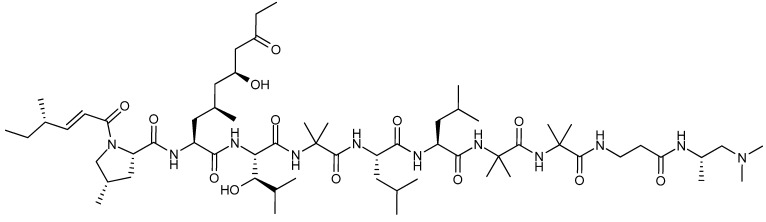
Leucinostatin A.

The peptide leucinostatin A (Figure 16) was first isolated in 1973 from *Purpureocillium lilacinum* (originally published as *Penicillium lilacinum* or *Paecilomyces lilacinus*) [132,133]. It was found active against a number of fungi and Gram-positive bacteria, as well as Ehrlich solid carcinoma of mice with an ED_50_ value of 1.6 mg/kg [132,134]. Furthermore leucinostatin A inhibited a long range of breast, melanoma, lung, ovary, colon and laryngeal cancers as well as eight leukemia cell lines with IC_50_ values between 4 nM and 12 µM [132,135,136]. Leucinostatin A inhibited growth of prostate cancer DU-145 cells *in vitro* and *in vivo* [137]. A natural analog leucinostatin A β di-*O*-glycoside displayed active against breast cancer BT-20 cell line, though not as potent as leucinostatin A [138].

## 4. Terpenoid-Derived Anticancer Compounds

Terpenoids form a third large and structurally diverse biosynthetic family of natural products derived from C_5_ isoprene units. Until now active anticancer terpenoids mostly belong to the sesqui- or diterpenoids [15].

**Figure 17:**
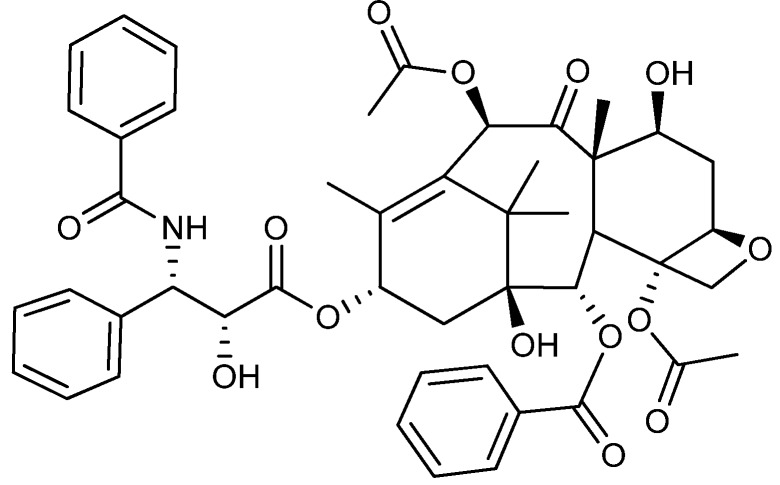
Taxol.

Taxol, also known as paclitaxel (Figure 17), is one of the best known anticancer drugs produced by fungi, though it originally was isolated from the bark of yew tree *Taxus brevifolia* [139]. Clinical development of taxol was delayed due to problems with production of large enough quantities of the compound. This problem was solved 20 years later when it was demonstrated that taxol was also produced by the fungus *Taxomyces andreanea* [140] and later including *P. raistrickii* [141].

Taxol was approved as anticancer drug against a wide range of tumors in the 1990s and is the first billion dollar drug against cancer [142,143]. Taxol functions by inducing cell cycle arrest in G_2_/M phase as well as apoptosis through a unique mode-of-action by promotion and stabilization of tubulin polymerization [144]. Today, taxol is routinely used to treat ovarian, breast and lung tumors as well as Kaposi’s sarcoma [145]. Besides its anticancer activity taxol displays antifungal activity as well [146].

**Figure 18:**
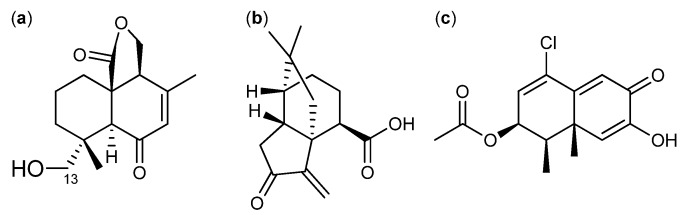
(**a**) Fudecadione A, (**b**) Terrecyclic acid A and (**c**) a novel unnamed chloro-trinoreremophilane sesquiterpene.

Many sesquiterpenes have been found active against cancer. Two of these are the drimane sesquiterpenes fudecadione A and B (Figure 18a) that were isolated in 2011 from *Penicillium* sp. BCC 17468. Fudecadione A was found active against human lung cancer NCI-H187, human breast cancer MCF-7, and human oral epidermoid carcinoma KB cell lines, with IC_50_ values of 24.9, 12.6 and 22.6 µM, respectively. Fudecadione B, on the other hand, was inactive against all three cancer call lines [147]. The activity of the two compounds suggests that the pharmacophore is located around the carbon at position 13, where fudecadione B is more branched [147].

Another active sesquiterpene is the antifungal terrecyclic acid A (Figure 18b), which was isolated from *A. terreus* in 1986 [148]. Terrecyclic acid A exhibit cytotoxic activity against human lung cancer NCI-H460, human breast cancer MCF-7, and human CNS cancer SF-268 cell lines with IC_50_ values of 10.6, 24.1 and 14.7 µM, respectively, and against leukemia in mice P-388 with LD_50_ values of 63-125 mg/kg [148,149].

In 2013 a novel and as yet unnamed chlorotrinoreremophilane sesquiterpene (Figure 18c) was isolated from *Penicillium* sp. PR19N-1 [150]. This novel sesquiterpene exhibited cytotoxic activity against human leukemia HL-60 and lung cancer A-549 cell lines with IC_50_ values of 11.8 and 12.2 µM, respectively [150].

**Figure 19:**
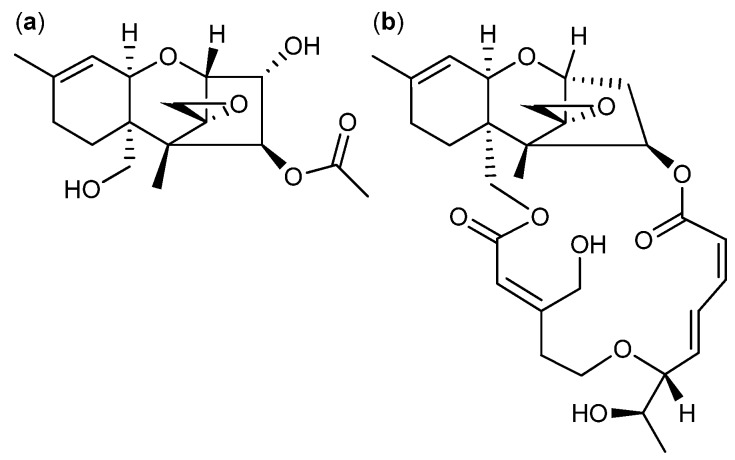
Trichothecenes (**a**) AETD and (**b**) 12′-Hydroxyroridin E.

A large group of mycotoxins called the trichothecenes cover more than 150 analogs and are mainly produced by a number of *Fusarium* spp. [151]. All the trichothecenes contain a sesquiterpenoid ring structure with an epoxide. The epoxide is often responsible for the cytotoxic activity by binding to the 60S ribosomal subunit in eukaryote cells thereby inhibiting protein synthesis [152,153]. Many of the trichothecenes exhibit cytotoxic activity against both fungi and cancer cell lines [154,155,156]. One of the more potent is AETD (Figure 19a) that inhibits HL-60, U-937, HeLa, MCF-7 and Hep-G2 cell lines with IC_50_ values of 10, 22, 45, 53 and 170 nM [157]. Other bioactive groups are the roridins where a macrocyclic ring is connected to the sesquiterpenoid unit. One of the more active compounds of this group is 12′-hydroxyroridin E (Figure 19b), which inhibits leukemia L-1210 with an IC50 value of 0.2 µM [158]. Another trichothecene called anguidine even entered clinical trials against cancer, but did not progress beyond phase II due to a lack of therapeutic efficacy [152,159].

**Figure 20:**
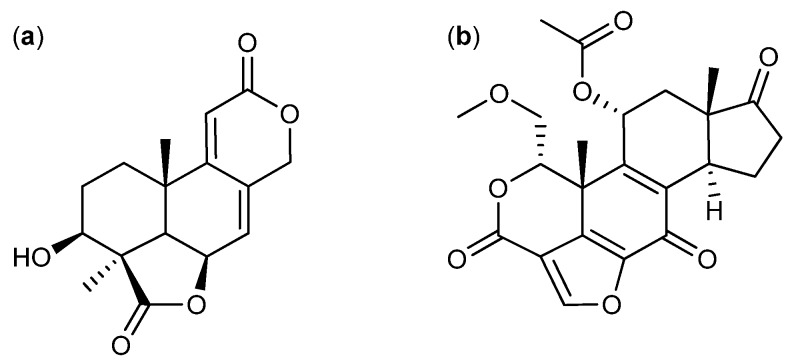
(**a**) Wentilactone B and (**b**) Wortmannin.

Wentilactone A and B (Figure 20a) were isolated from *A. wentii* in 1980 [160]. In 2012 it was reported that wentilactone B induced apoptosis and inhibited proliferation in human hepatoma cancer cell line SMMC-7721, with an IC_50_ value of 19 µM [161].

The antifungal compound wortmannin (Figure 20b) is produced by *T. wortmannii* originally published as *P. wortmannii* [162] or a related species. Wortmannin inhibit the activity of leukemia HL-60 and K-562 cell lines with IC_50_ values of 30 and 25 nM, respectively [163,164], including the breast cancer MCF-7 cell line that was inhibited by 51.3% after 48 h with a concentration of 25 nM [165]. In human pancreatic cancer cells lines PK1 and PK8 wortmannin induces apoptosis both *in vitro* and *in vivo* [166,167]. Finally, wortmannin has been shown to inhibit proliferation in lung cancer cell lines KNS-62 and Colo-699 both *in vitro* and *in vivo*, with IC_50_ values between 100 and 200 nM [168].

**Figure 21:**
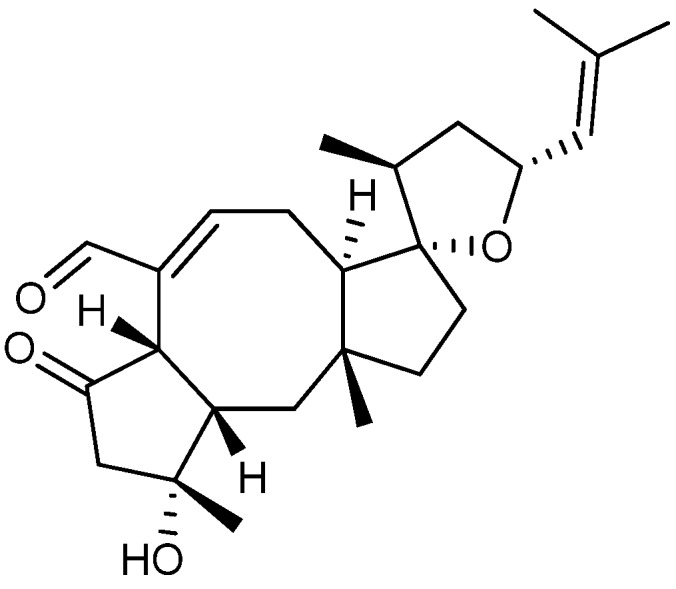
Ophiobolin A.

The ophiobolins are sesterterpenes mainly isolated from the fungal genus *Bipolaris* [169,170], but are also found in *Aspergillus* [171], *Sarocladium* [172,173], and *Drechslera* [174]. Isolation of ophiobolins from *Aspergillus* spp. misidentified as *A. ustus* were later ascribed to the three different species: *A. calidoustus*, *A. insuetus*, and *A. keveii* from the *Aspergillus* section *Usti* [69]. Ophiobolin A (Figure 21) exhibit inhibitory activity against cancer cell lines includes lung cancer A-549, colon cancer HT-29, melanoma Mel-20, leukemia P-388, and ovarian cancer OVCAR-3 with IC_50_ values of 0.1, 0.1, 0.1, 0.06 and 0.3 µM, respectively [175,176]. In 2012 the novel ophiobolin O was discovered and it was found to inhibit breast cancer MCF-7 and leukemia P-388 cell lines with IC_50_ values of 17.9 and 4.7 µM, respectively [177,178]. Most recently in 2013 the novel 3-anhydro-6-hydroxyophiobolin A was isolated and found active against lung cancer (HepG2) and leukemia (K-562) cell lines with IC_50_ values of 6.5 and 4.1 µM, respectively [179]. Besides anticancer activity the ophiobolin family show antifungal activity against a wide range of fungi [180,181].

## 5. Anticancer Natural Products of Mixed or Unresolved Biosynthetic Origin

**Figure 22:**
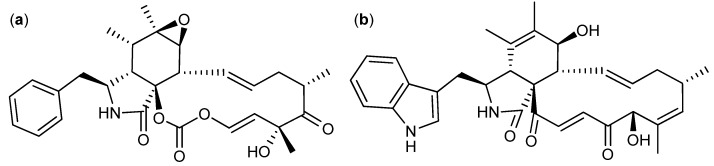
(**a**) Cytochalasin E and (**b**) Chaetoglobosin B.

Filamentous fungi are capable of producing secondary metabolites of mixed biosynthetic origin. This includes both compounds such as meroterpenoids that comprise a huge class of compounds that integrate a polyketide part with a terpenoid part [182], in addition to the cytochalasins [183] and chaetoglobosins that are biosynthesized by incorporation of amino acids into a core polyketide part. The cytochalasins contains a phenylalanine coupled to the polyketide chain where the chaetoglobosins have an tryptophan moiety [183,184]. Both the cytochalasins and the chaetoglobosins exhibit antifungal activities against a broad range fungal species [56,185,186]. The cytochalasins are produced by many fungal genera including *Aspergillus*, *Hypoxylon*, *Metarrhizium*, *Zygosporium*, *Hypocrella* and *Phoma* [90,187,188,189,190]. Many of the cytochalasins have shown inhibitory activities towards lung cancer A-549. One of the more potent is cytochalasin E (Figure 22a) which inhibited human ovarian A-2780S, human colon HCT-116 and SW-620, and lung A-549 cancer as well as human leukemia P-388 with IC_100_ values of 0.02, 1.0, and 0.2 µg/ml and IC_50_ values of 0.006 and 0.09 µM, respectively [191,192].

The chaetoglobosins were originally isolated from *Chaetomium globosum* in 1973 [184]. Several chaetoglobosins have been isolated over the years from *P. discolor* and *P. expansum* among others [1] and many of them showed activity against cancer cell lines. Of the more potent ones was chaetoglobosin B (Figure 22b) that inhibited human breast BC cancer cell line with an IC_50_ value of 3.0 µM [193] and chaetoglobosin D that inhibited adenocarcinoma KKU-100 and KKU-OCA17 cancer cell lines with IC_50_ values of 3.4 and 12.2 µM, respectively [193]. Chaetoglobosin U showed activity as well against KB tumor cell line with an IC_50_ value of 16.0 µM [194], and chaetoglobosin X with activity against murine hepatic cancer H-22 cell line with an IC_50_ value of 7.5 µM [195].

**Figure 23:**
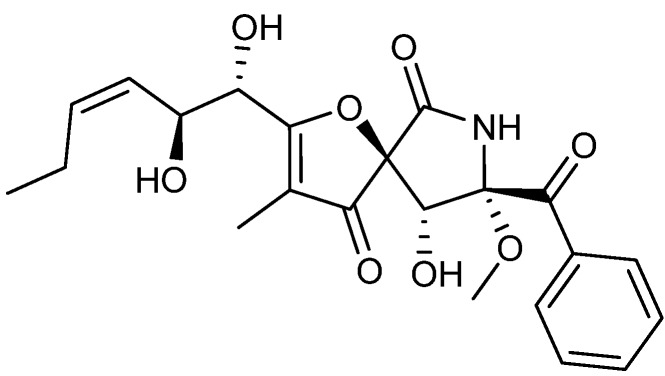
Pseurotin A.

Another group of compounds with mixed a biosynthetic pathway are the pseurotins (Figure 23) containing phenylalanine coupled to a polyketide and with a spiro ring structure. The pseurotins were first isolated from the bacteria *Pseudeurotium ovalis* and later from the fungus *A. fumigatus* [196,197]. In 2012 four pseurotin analogs were proven active against a human breast cancer cell line MCF-7 with the more active pseurotin D inhibiting MCF-7 with an IC_50_ value of 15.6 µM [198].

**Figure 24:**
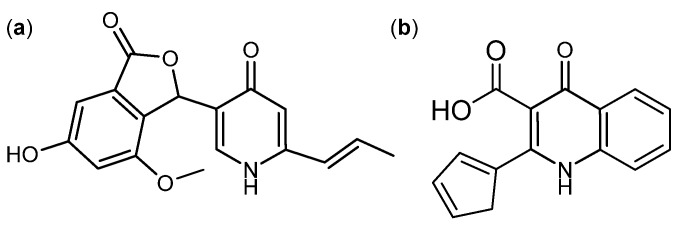
(**a**) Penicidone B and (**b**) Penicinoline.

The γ-pyridone alkaloids, penicidone A–C (Figure 24a) were isolated from an endophytic *Talaromyces* sp. The biosynthesis of penicidones probably happens through the polyketide pathway similar to penisimplicissin (Figure 24b), but with a unique introduction of nitrogen [199]. The penicidones were showed to be active against human colon cancer SW1116, human AML (K-562), and human cervical cancer (KB and HeLa) cell lines with penicidone B being the more potent analog. Penicidone B inhibited the four cancer cell lines with IC_50_ values of 54.2 µM (SW1116), 21.1 µM (K-562), 29.6 µM (KB), and 35.2 µM (HeLa) [199]. Two other γ-pyridone alkaloids called penicinoline (Figure 24b) and methylpenicinoline were isolated from an endophytic *Penicillium* sp. and *Auxarthron reticulatum* in 2010 and 2011, respectively [200,201]. Penicinoline and methylpenicinoline were shown to inhibit the lung cancer HepG2 cell line with IC_50_ values of 13.2 and 11.3 µM, respectively [202].

**Figure 25:**
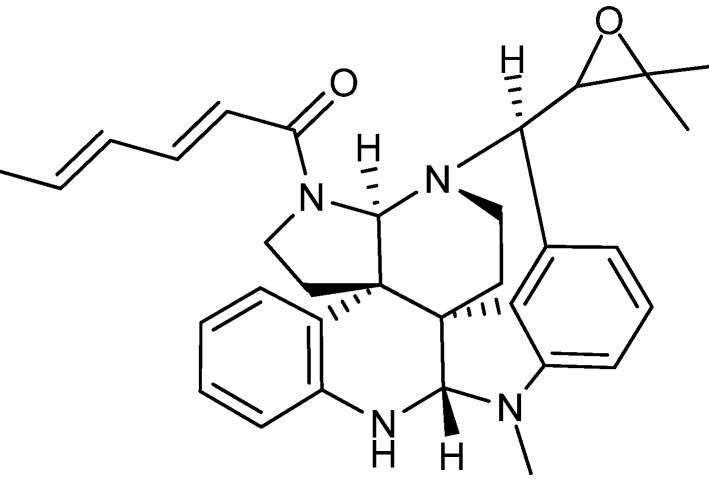
Communesin B.

Communesins are fungal metabolites produced in *P. marinum*, *P. expansum*, *P*. *buchwaldii* and *P. rivulorum* [96,203,204,205]. The group is of highly mixed biosynthetic origin with structures combining a nitrogen-containing part, a polyketide chain and isoprene. Communesin B (Figure 25) displays the highest activity of them all against leukemia P-338, U-937, THP-1, NAMALWA, MOLT-3 and SUP-B15 cell lines with ED_50_ values of 0.5, 10.4, 11.4, 9.9, 8.1 and 7.2 µg/mL, respectively [206,207].

**Figure 26:**
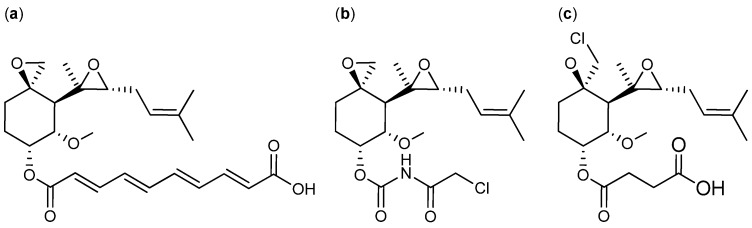
(**a**) Fumagillin, (**b**) Synthetic analog TNP-470 and (**c**) Ligerin.

The antibiotic compound fumagillin (Figure 26a) has been one of the more potent and interesting compounds associated with anticancer activity produced by fungi. Fumagillin, classified as a meroterpenoid, was first isolated from *A. fumigatus* in 1951 [208] and further produced by *P. scabrosum* [209]. The antitumor activity of fumagillin is caused by its potent angiogenesis-inhibiting effect. Unfortunately, fumagillin had some unpleasant side effects and consequentially investigations into structural analogs have been conducted [210]. The synthetic fumagillin analog TNP-470 (Figure 26b) contains an amine and chlorine in the side chain, which led to a highly increased angiogenesis—inhibiting effect. Furthermore, TNP-470 is less toxic to normal cells compared to fumagillin [210,211]. *In vitro* and *in vivo* tests showed that TNP-470 inhibited tumor growth in prostate (PC-3) and breast cancer (MDA-MB-231). TNP-470 entered preclinical trails in 1992 and phase I and II in 2000 but did not progress into phase III, due to a very short systemic half-live and observed neurotoxicity in patients [212,213,214]. Recently, in 2013, a novel natural fumagillin analog namely ligerin (Figure 26c) was isolated from a marine-derived *Penicillium* sp. [215]. Ligerin inhibited lung cancer POS1 cell line with an IC_50_ value of 117 nM [215].

**Figure 27:**
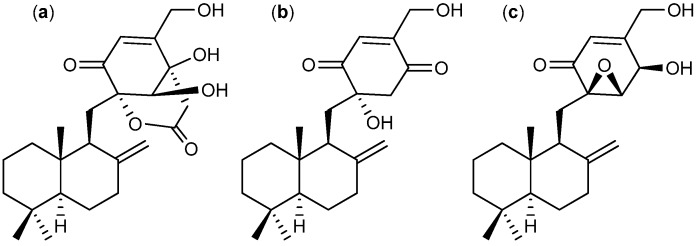
(**a**) Purpurogemutantin, (**b**) Purpurogemutantidin and (**c**) Macrophorin A.

A strain so far identified as *Talaromyces purpurogenus* (earlier *P. purpurogenum*) is known to produce another group of meroterpenoids namely purpurogemutantin (Figure 27a), purpurogemutantidin (Figure 27b), and the known antifungal macrophorin A (Figure 27c) [216,217]. All three compounds inhibit human leukemia K-562 and HL-60, human cervical cancer HeLa, human gastric adenocarcinoma BGC-823 and human breast cancer MCF-7 cell lines. Purpurogemutantidin (Figure 27a) was the more potent, with IC_50_ values of 0.9, 2.4, 16.6, 31.0, and 26.3 µM, respectively. The only exception is macrophorin A that displayed higher activity towards the HL-60 cell line with an IC_50_ value of 0.9 µM [216].

**Figure 28:**
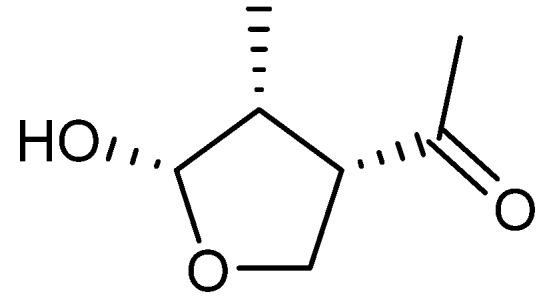
Botryodiplodin (PSX-1).

Botryodiplodin (PSX-1), a small antifungal [218] compound of unresolved biosynthetic origin (Figure 28), was isolated from *T. stipitatus* (originally named *P. stipitatum*) [219] and exhibits activity against Ehrlich ascites carcinoma, leukemia L-5178, Sarcoma 37, and cervical cancer Hela cell lines with a median effective dose (ED_50_) values of approximately 1 µg/mL [219,220].

**Figure 29:**
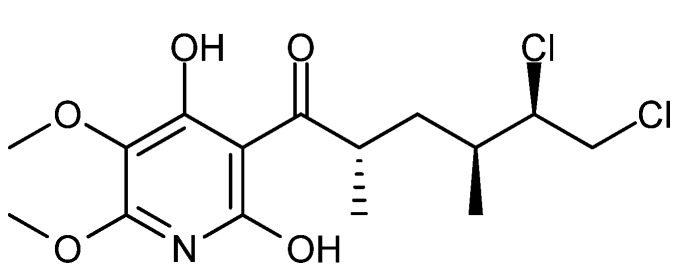
Atpenin A5.

The atpenins A4, A5 and B (Figure 29) of unresolved biosynthetic pathway were first isolated from *P. atramentosum* in 1988 and shown to possess antifungal activities [221]. Twenty years later the first three atpenins were isolated again from the same fungi along with two new analogs, NBRI23477 A and B [222]. The activities of the five atpenins were examined individually against human prostate cancer cell line DU-145 and in a stromal co-cultured assay. Growth of the DU-145 cells was inhibited more effectively in the co-cultured setup, with atpenin A5 being the more potent analog with an IC_50_ value of 0.02 µg/mL [222]. All the reviewed compounds are summarized in Table 1 with their individual organisms of origin, biosynthetic pathways, target cancer cells, IC_50_ (if available), and whether the compounds exhibit antifungal activity too.

**Table 1:** List of fungal anticancer natural products primarily produced by *Aspergillus*, *Penicillium* and *Talaromyces* summarizing producing organisms, biosynthetic origin, target cancer cells, IC_50_ (if available), and if the compounds exhibit antifungal activity.

Species (original published)	Compound	Type	Target cell	IC_50_	Anti-fungal (+/−)
*T. pinophilus* [1] (*P. pinophilum* [48])	3-O-methylfunicone [50,51,52,53]	Polyketide	HeLa	-	+ [48,49]
			MCF-7	-	
			A-375P	-	
			A-375M	-	
*A. nidulans* [46]	Asperlin [47]	Polyketide	HeLa	-	−
*A. flavus* [95] *Penicillium* spp. [96]	Asperphenamate	NPR	-	-	−
*A. flavus* [95] *Penicillium* spp. [96]	BBP [99]	NPR	MCF-7	3.0 µM	
			T47D	4.7 µM	
			MDA-MB231	5.2 µM	
			BEL-4702	12.7 µM	
			A-549	15.1 µM	
			HeLa	17.0 µM	
			HL-60	18.3 µM	
*Aspergillus* sp. [131]	Acetylapoaranotin [131]	NPR	HCT-116	13.8 µM	−
			AGS	12 µM	
			A-549	2 µM	
			MCF-7	10 µM	
*P. atramentosum* [222]	Atpenin A5 [222]	Unresolved biosynthetic origin	DU-145 (co-culture)	0.02 µg/mL	+ [221]
*A. puniceus* [69] *A. turkensis* [69] *A. pseudoustus* [69] *A. ustus* [70]	Austocystin D [71,72]	Polyketide	SR	16 nM (GI_50_)	−
			U-87	4946 nM (GI_50_)	
			MCF-7	<1 nM (GI_50_)	
			MDA-MB-231	549 nM (GI_50_)	
			PC-3	3 nM (GI_50_)	
			SW-620	27 nM (GI_50_)	
			HCT-15	42 nM (GI_50_)	
			MX-2	3358 nM (GI_50_)	
*P. brevicompactum* [20] *P. paneum* [223] *T. stipitatus* [1] (*P. stipitatum* [220])	Botryodiplodin [219,220]	Unresolved biosynthetic origin	EAC	0.6 µg/mL (ED_50_)	+ [218]
			L-5178	0.8 µg/mL (ED_50_)	
			Sarcoma 37	1.5 µg/mL (ED_50_)	
			HeLa	2.0 µg/mL (ED_50_)	
*P. brefeldianum* [39]	Brefeldin A [42,43,44,45]	Polyketide	HL-60	35.7 nM	+ [39,40]
			KB	32 nM	
			HeLa	6.4 nM	
			MCF-7	7.1 nM	
			SPC-A-1	3.6 nM	
			BC-1	40 nM	
			NCI-H187	110 nM	
*Cladosporium cladosporioides* [82]	Calphostin C [82,83]	Polyketide	HeLa S3	0.2–8.5 µM	−
			MCF-7	0.2–2.7 µM	
			ALL	-	
*Chaetomium globosum* [184] *P. discolor* [1] *P. expansum* [1]	Chaetoglobosin B [193]	Mixed biosynthetic origin	BC	3.0 µM	+ [56,186]
*Chaetomium globosum* [184] *P. discolor* [1] *P. expansum* [1]	Chaetoglobosin D [193]	Mixed biosynthetic origin	KKU-100	3.4 µM	
			KKU-OCA17	12.2 µM	
*Chaetomium globosum* [184] *P. discolor* [1] *P. expansum* [1]	Chaetoglobosin U [194]	Mixed biosynthetic origin	KB	16.0 µM	
*Chaetomium globosum* [184] *P. discolor* [1] *P. expansum* [1]	Chaetoglobosin X [195]	Mixed biosynthetic origin	H-22	7.5 µM	
*Chaetomium globosum* [57,58,59,60,61,63,64] *T. pinophilus* [1]	11-epichaetomugilin I [62]	Polyketide	P-388	0.7 pM	+ [56]
			HL-60	1.0 pM	
			L-1210	1.6 pM	
			KB	1.2 pM	
*P. chrysogenum* [74] *P. rubens* [73] (*P. terrestre* [76])	Chloctanspirone A [76]	Polyketide	HL-60	9.2 µM	−
			A-549	39.7 µM	
*P. buchwaldii* [96] *P. marinum* [1] *P. expansum* [204] *P. rivulorum* [205]	Communesin B [206,207]	Mixed biosynthetic origin	P-388	0.5 µg/mL (ED_50_)	−
			U-937	10.4 µg/mL (ED_50_)	
			THP-1	11.4 µg/mL (ED_50_)	
			NAMALWA	9.9 µg/mL (ED_50_)	
			MOLT-3	8.1 µg/mL (ED_50_)	
			SUP-B15	7.2 µg/mL (ED_50_)	
*Mariannaea elegans* (*Spicaria elegans* [192]) *A. clavatus* [190] *A. rhizopodus* [90] *Hypoxylon terricola* [187]	Cytochalasin E [191,192]	Mixed biosynthetic origin	A-2780	0.02 µg/mL (IC_100_)	+ [185]
			HCT-116	1.0 µg/mL (IC_100_)	
			SW-620	0.2 µg/mL (IC_100_)	
			A-549	0.006 µM	
			P-388	0.09 µM	
*A. striatus* (*Emericella striata* [128]) *Cladorrhinum* sp.	Emestrin A [129]	NPR	HL-60	83.5 nM	+ [127]
*A. striatus* (*Emericella striata* [128]) *Cladorrhinum* sp.	Emestrin C [130]	NPR	DU-145	9.3 nM	
*Penicillium* sp. BCC 17468 [147]	Fudecadione A [147]	Terpene	MCF-7	12.6 µg/mL	−
			KB	22.3 µg/mL	
			NCI-H187	24.9 µg/mL	
*A. fumigatus* [208] *P. scabrosum* [209]	Fumagillin [210]	Mixed biosynthetic origin	Angiogenesis inhibitor	-	−
	TNP-470 [210,211,212,213,-214]	Synthetic	PC-3	-	
			MDA-MB-231	-	
*Penicillium* sp. [215]	Linerin [215]	Mixed biosynthetic origin	POS1	117 nM	
*A. fumigatus* [101,102] *A. fischeri* [103]	Fumitremorgin C [111,113,114]	NPR	P-388	3.9 µg/mL (ED_50_)	+ [224]
*A. fumigatus* [101,102] *A. fischeri* [103]	12,13-dehydroxyfumitremorgin C [112]	NPR	U-937	1.8 µM	
			PC-3	6.6 µM	
*A. fumigatus* [122] *D. cejpii* [123]	Gliotoxin [112,125,126]	NPR	MCF-7	985 nM	+ [124]
			T47D	365 nM	
			BT-474	102 nM	
			ZR75-1	158 nM	
			MDA MB231	38 nM	
			MDA MB435	87 nM	
			U-937	0.2 µM	
			PC-3	0.4 µM	
*P. griseofulvum* [225]	Griseofulvin [2,3,4,226,227]	Polyketide	HeLa	20 µM	+ [226,228]
			SCC-114	35 µM	
	GF-15 [5]	Synthetic	SCC-114	0.9 µM	
*Hypocrella bambusae*(*Shiraia bambusicola* [84,85])	Hypocrellin D [85]	Polyketide	Bel-7721	1.8 µg/mL	+ [229]
			A-549	8.8 µg/mL	
			Anip-973	38.4 µg/mL	
	Synthetic analog [86]	Synthetic	MCF-7 (PDT)	-	
*Purpureocillium lilacinum* [133] (*P. lilacinum* [132])	Leucinostatin A [132,135,136,137,138]	NPR	Ehrlich	1.6 mg/kg (LD_50_)	+ [132,134]
			L-1210	410.3 nM (IC_100_)	
			BT-20	2.3 nM	
			MCF-7	4 nM	
			G-361	7 nM	
			HT-144	6 nM	
			A-549	16 nM	
			A-427	59 nM	
			SK-MES-1	12 µM	
			Caov-3	17 nM	
			Caov-4	53 nM	
			SKOV-3	1,236 nM	
			HT-29	119 nM	
			LoVo	114 nM	
			HEp-2	40 nM	
			HL-60	12 nM	
			HSB-2	5 nM	
			K-562	6 nM	
			KG-1	46 nM	
			RPMI-1788	158 nM	
			SKW-6.4	6 nM	
			U-937	319 nM	
			Raji	11 nM	
*A. parasiticus* [65,66] *A. nidulans* [67,68]	Norsolorinic acid [67,68]	Polyketide	T-24	10.5 µM	−
			MCF-7	12.7 µM	
*A. amoenus* [115]	Notoamides [116,230]	NPR	HeLa/L-1210	22–52 µg/mL	−
*Bipolaris *sp. [169,170] *A. calidoustus* [69] *A. insuetus* [69] *A. keveii* [69] (*A. ustus* [171]) *Sarocladium oryzae* [173] (*Cephalosporium caerulens* [172] (*D. maydis* [174] *D. sorghicola* [174]	Ophiobolin A [175,176]	Terpene	A-549	0.1 µM	+ [180,181]
			HT-29	0.1 µM	
			Mel-20	0.1 µM	
			P-388	0.06 µM	
			OVCAR-3	0.3 µM	
*Bipolaris oryzae* [179]	3-anhydro-6-hydroxyophiobolin A [179]	Terpene	HepG2	6.5 µM	
			K-562	4.1 µM	
*Aspergillus section Usti* [69] (*A. ustus* [177])	Ophiobolin O [177,178]	Terpene	MFC-7	17.9 µM	
			P-388	4.7 µM	
*Talaromyces* sp. (*Penicillium* sp.) [199]	Penicidone B [199]	Mixed biosynthetic origin	SW1116	54.2 µM	−
			K-562	21.1 µM	
			KB	29.6 µM	
			HeLa	35.2 µM	
*Penicillium* sp. [200] *Auxarthron reticulatum* [201]	Penicinoline [202]	Mixed biosynthetic origin	HepG2	13.2 µM	−
*Penicillium*sp. [200] *Auxarthron reticulatum* [201]	Methyl-penicinoline [202]	Mixed biosynthetic origin	HepG2	11.3 µM	
*T. pinophilus* [1] (*P. simplicissimum* [54])	Penisimplicissin [55]	Polyketide	HL-60	−6.7 (log10 GI_50_)	−
			CCRF-CEM	−5.8 (log10 GI_50_)	
*Phaeosphaeria* sp. [87]	Phaeosphaerin B [87]	Polyketide	PC-3	2.4 µM	−
			DU-145	9.5 µM	
			LNCaP	2.7 µM	
*A. ustus* [117]	Phenylahistin [118]	NPR	A-431	0.22 µM	−
			A-549	0.30 µM	
			HeLa	0.20 µM	
			K-562	0.19 µM	
			P-388	0.33 µM	
			MCF-7	3.7 µM	
			TE-671	0.18 µM	
*A. taichungensis* [79]	Prenylterphenyllin A [79]	Polyketide	A-549	8.3 µM	−
			HL-60	1.5 µM	
*A. candidus* [77,78]	4”-deoxyisoterprenin [78]	Polyketide	KB3-1	6.2 µM	
*A. taichungensis* [79]	Prenylcandidusin B [79]	Polyketide	P-388	1.6 µM	
*Pseudeurotium ovalis* [196] *A. fumigatus* [197]	Pseurotin D [198]	Mixed biosynthetic origin	MFC-7	15.6 µM	−
*T. purpurogenus* mutant BD-1-6 [1] (*P. purpurogenum* [216])	Purpurogemutantidin [216]	Mixed biosynthetic origin	K-562	0.9 µM	+ [217]
			HeLa	16.6 µM	
			BGC-823	31.0 µM	
			MCF-7	26.3 µM	
*T. purpurogenus* mutant BD-1-6 [1] (*P. purpurogenum* [216])	Macrophorin A [216]	Mixed biosynthetic origin	HL-60	0.9 µM	
*A. parasiticus* [231]	Sequoiamonascin A [231]	Polyketide	MCF-7	1%	−
			NCI-H460	1%	
			SF-268	2%	
				Percent cell growth compared to untreated cells at 10 µM	
*P. solitum* [20,25] (*P. brevicompactum* [18]) *P. hirsutum* [20] (*P. citrinum* [19])	Compactin [28]	Polyketide	AML	2.6 µM (IC_100_)	+ [18]
*A. terreus* [26] *Monascus* sp. [27]	Lovastatin [29,30,31,32]	Polyketide	OVHS-1	39 µM	+ [22,23,24]
			Calu-1	3 µM	
			H-460	3 µM	
			A-549	10 µM	
			H-441	30 µM	
			Ovca-432	2 µM	
			A-2780	3 µM	
			Hey	3 µM	
			Ovca-429	5 µM	
			HOC-7	10 µM	
			DOV-13	11 µM	
			Skov-3	21 µM	
			A-2780-ADR	21 µM	
			A-2780-CIS	22 µM	
			MCF-7	1.7 µM	
			HepG2	2.7 µM	
			HeLa	1.5 µM	
			AML	-	
	Simvastatin [1,20,29,33,34]	Synthetic	DLRP	0.9 µM	+ [23,24]
			H-1299	1.3 µM	
			HT-144	1.0 µM	
			MI-14	0.8 µM	
			SK-MEL-28	0.8 µM	
			BT-474A	4.2 µM	
			SKBR-3	2.2 µM	
			MDA-MB-453	5.4 µM	
			BT-20	1.7 µM	
			AML	-	
*A. ochraceus* [106] *A. westerdijkiae* [107]	Stephacidin B [106]	NPR	PC-3	0.4 µM	−
			LNCaP	0.06 µM	
			A-2780	0.3 µM	
			A-2780/DDP	0.4 µM	
			A-2780/Tax	0.3 µM	
			HCT-116	0.5 µM	
			HCT-116/mdr+	0.5 µM	
			HCT-116/topo	0.4 µM	
			MCF-7	0.3 µM	
			SKBR-3	0.3 µM	
			LX-1	0.4 µM	
*Taxomyces andreanea* [140] *P. raistrickii* [141]	Taxol [139,142,143,144,145]	Terpene	Clinical use: Ovarian tumors Breast tumors Lung tumors Kaposi’s sarcoma	-	+ [146]
*A. terreus* [148]	Terrecyclic acid A [148,149,232]	Terpene	NCI-H460	10.6 µM	+ [148]
			MCF-7	24.1 µM	
			SF-268	14.7 µM	
			P-388	63-125 mg/kg (LD_50_)	
*A. terreus* [37]	Terrein [38]	Polyketide	MCF-7	1.1 nM	+ [36]
			PANC-1	9.8 µM	
			HepG2	66.8 µM	
*Fusarium *spp. [151] *Isaria japonica* [157]	4-Acetyl-12,13-epoxy-9-trichothecene- 3,15-diol (AETD) [157]	Terpene	HL-60	10 nM	+ [154]
			U-937	22 nM	
			HeLa	45 nM	
			MCF-7	53 nM	
			Hep-G2	170 nM	
*Fusarium *spp. [151] *Myrothecium roridum* [158]	12’hydroxyroridin E [158]	Terpene	L-1210	0.2 µM	
			BC-1	0.9 µM	
			NCI-H187	1.5 µM	
*P. citrinum* [80]	Tricitrinol B [80]	Polyketide	HL-60	3.2 µM	−
			HCT-116	4.8 µM	
			KB	3.9 µM	
*A. fumigatus* [104,105,108,109]	Ds2-tryprostatin B [108]	NPR	NCI-H-522	15.8 µM (GI_50_)	−
			MCF-7	15.9 µM (GI_50_)	
			PC-3	11.9 µM (GI_50_)	
*A. fumigatus* [104,105,108,109]	18-oxotryprostatin A [110]	NPR	A-549	1.3 µM	
*A. fumigatus* [104,105,108,109]	Spirotryprostatin E [109]	NPR	MOLT-4	3.1 µM	
			HL-60	2.3 µM	
			A-549	3.1 µM	
*Penicillium* sp. [150]	Unnamed chloro-trinoreremophilane sesquiterpene [150]	Terpene	HL-60	11.8 µM	−
			A-549	12.2 µM	
*A. wentii* [160]	Wentilactones [161]	Terpene	SMMC-7721	19 µM	+ [233]
*T. wortmannii* [1] (*P. wortmannii* [162]) Or similar species.	Wortmannin [163,164,165,166,167,168]	Terpene	HL-60	30 nM	+ [162]
			K-562	25 nM	
			KNS-62	100–200 nM	
			Colo-699	100–200 nM	
*P. chrysogenum* [1] *D. cejpii* [90] (*D. albus* [90])	Xanthocillin X [89,91,92]	NPR	Ehrlich ascites carcinoma-mouse strain	40 mg/kg (LD_50_)	+ [89]
			K-562	0.4 µg/mL	
			HeLa	1.2 µg/mL	
			MCF-7	12 µg/mL	
			HepG2	7 µg/mL	
			NCI-H460	10 µg/mL	
			Du-145	8 µg/mL	
			MDA-MB-231	8 µg/mL	
*Aspergillus* sp. [93]	BU-4704 [93]	NPR	HCT-116	0.6 µg/mL	
			B16-F10	4.3 µg/mL	
*P. chrysogenum* [1]	Xanthocillin X di-methoxy [94]	NPR	HepG2	0.2 µg/mL	
			MCF-7	0.4 µg/mL	
			KB	0.4 µg/mL	

## 6. Conclusions

In this article we have reviewed 50 compounds or compound families with anticancer and often also antifungal activities, primarily produced by *Aspergillus*, *Penicillium* and *Talaromyces*. Mycologists predict that less than 10% of all fungal species have been isolated so far, indicating a huge potential for further discovery of novel bioactive chemical scaffolds, if these fungi can be cultured in the laboratory. New strategies such as the application of epigenetic modifiers may help to uncover the full biosynthetic potential of fungi and other microorganisms. Development and improvement of screening methods and assays will further assist revealing new bioactivities of already known compounds. Additionally, ongoing progress in fast dereplication, including improvement of the performance of mass spectrometers and high resolution of UHPLC chromatograms, will ensure that database searches will lead to fewer possible candidates thereby advancing the drug discovery process. Altogether the future seems promising for discovery of many more bioactive small molecules to be used either as scaffolds for: (i) diversity oriented synthesis, or (ii) as a starting point for cloning and engineering of whole biosynthetic gene clusters towards novel engineered bioactive natural products.
